# Predictive performance of the estimating equations of renal function in Sri Lankan subjects

**DOI:** 10.1186/s13104-019-4692-3

**Published:** 2019-10-11

**Authors:** Ranga Migara Weerakkody, Mohammed Hussain Rezvi Sheriff

**Affiliations:** 1 Department of Nephrology, Dialysis and Transplantation, Teaching Hospital, Jaffna, Sri Lanka; 2 Department of Clinical Medicine, Faculty of Medicine, General Sir John kotelawala Defence University, Ratmalana, Sri Lanka

**Keywords:** Glomerular filtration rate, MDRD, CKD-EPI, Sri Lanka, Creatinine clearance

## Abstract

**Objectives:**

This study validates two popular predictive equations of renal function firstly, Modifications of Diet in Renal Disease and secondly, Chronic Kidney Disease Epidemiology Collaboration equations for Sri Lankan cohort. We used data of the patients referred to Renal Research lab of University of Colombo for creatinine clearance measurement.

**Results:**

Predictive performances varied with the gender. Creatinine clearance and predicted renal functions were compared. Both fared unsatisfactorily with R^2^ ranging from 0.632 to 0.652, and overestimated renal function by 6–15%. The proportion chronic kidney disease staging 1 and 2 returned by Chronic Kidney Disease Epidemiology Collaboration equation showed significant difference, in females. Modifications of Diet in Renal Disease equation significantly under-estimated advanced chronic kidney disease in females. Chronic Kidney Disease Epidemiology Collaboration equation had better accuracy. The study sample had more females, Asian and lower body size and better renal functions than historic cohorts. Thai and Pakistani studies show both equations and their Asian adaptations fare poorly. Chronic kidney disease stages differ significantly with the equation used. Predictive equations have fared unsatisfactorily by overestimating renal functions. We recommend further studies using gold standards of measuring renal function.

## Introduction

The prevalence and incidence of renal diseases is continuously increasing in US and other countries [1–4], and similar trend is expected in Sri Lanka as well. To level off these incident rates, various initiatives, such as the Kidney Disease Outcomes Quality Initiative (K/DOQI), have provided physicians with guidelines to optimize the care of patients with chronic kidney disease (CKD). These guidelines emphasize the need to assess kidney function using predictive equations rather than serum creatinine alone [1]. It is also highlighted the need of the equations to be validated in large samples of subjects, in particular that they should be tested in non-US populations and in individuals with mild decrease in kidney function or normal renal functions [5]. Validation of the predictive formulas is also particularly important for patients aged 65 and older, who by far have the highest incident rates of end stage renal disease (ESRD) [4, 6, 7].

Estimated glomerular filtration rate (eGFR) is an important parameter in clinical practice as well. The renal dose adjustments of the drugs are based on CKD stages, which in turn dependent of eGFR. This makes predictive performance of the equations extremely important, to prevent over or under dosing of the drugs.

The formulae most widely used to estimate kidney function, as well as being recommended in adults by the K/DOQI guidelines, [5] are the Cockcroft–Gault (CG) formula [8] and the recently developed [9] and later simplified [10] Modification of Diet in Renal Disease (MDRD) formula. The CG formula is an estimate of creatinine clearance originally developed in a population of 236 Canadian patients, 209 of which were male. The MDRD formula have been developed as an estimation of ^125^I-Iothalamate renal clearance based GFR measurement in a population of 1628 patients with previously diagnosed CKD [9–11]. The Chronic Kidney Disease Epidemiology Collaboration (CKD EPI) formula was published in May 2009. It was developed in an effort to create formula more accurate than the MDRD formula, especially when actual GFR is greater than 60 ml/min/1.73 m^2^. Researchers pooled data from multiple studies to develop and validate this new equation. The CKDEPI equation performed better than the MDRD equation, especially at higher GFR, with less bias and greater accuracy. National Health and Nutrition Examination Survey (NHANES) data, the median GFR was 94.5 ml/min/1.73 m^2^ vs. 85.0 ml/min/1.73 m^2^, and the prevalence of chronic kidney disease was 11.5% versus 13.1% [12], using the two equations.

## Main text

### Methodology

This is a study conducted retrospectively. The data source is from Renal Research Lab (RRL) of the Department of Clinical Medicine, Faculty of Medicine, University of Colombo, where patients’ height, weight, gender, age, serum creatinine and creatinine clearance are recorded. Ethical clearance was obtained through the Review Committee of National Hospital of Sri Lanka.

The creatinine measurements of the lab, is regularly validated against standard strength solutions, and have shown no deviations around the levels of clinical significance. Jaffe’s method uses sodium 2,4,6 trinitrophenolate and then spectrophotometry was the method of creatinine estimation. The inward health personnel supervised the urine collection at all times. In catheterized patients, urine was directly collected to the collection bottle. Unless contra-indicated, every subject was asked to keep an intake of 2.5–3.5 l during collection period, and to stick to their usual dietary habits. The urine bottles were transported to RRL immediately after the collection process, where the transportation time was less than half an hour.

We included all patients aged more than 18 years in the study. We excluded records of all patients with acute renal failure, rhabdomyolysis, aged < 18, BMI < 17 kg/m^2^, BMI > 40 kg/m^2^, pregnancy and history of muscular disorders from the study.

We adopted abbreviated MDRD equation from the original work [9] and CKD EPI from original work of authors [13]. We corrected creatinine clearance for BSA, using the Dubois’ formula [14] and then, adjusted for over estimation by multiplying from 0.81 [9] (please see Additional file 1: Table S1 for the equations). The bias of the measurement was defined as the mean of the difference between estimated and measured GFR, while precision is defined as the standard deviation of the bias. We constructed Combined Root Mean Square (CRMSE) using the formula of (bias^2^ + precision^2^)^0.5^. The recorded data was analyzed using SPSS 21.0 statistical program.

### Results

#### Basic characteristics

Study population consisted of 475 subjects (n = 475), of which 212 were males (44.6%).

Table 1 shows basic characteristics of the study group.

**Table 1:** Summary of basic characteristics of the study population

Characteristic	Males	Females
Age (year)	42.5 (16.8)	44.9 (13.8)
Height (m)	159.3 (9.5)	149.2 (8.2)
Weight (kg)	61.5 (12.2)	54.7 (11.7)
Body surface area (m^2^)	1.63 (0.18)	1.48 (0.16)
Body mass index (kg/m^2^)	24.3 (4.6)	24.6 (5.2)
Creatinine clearance (ml/min)	69.3 (32.3)	76.8 (34.5)
eGFR–MDRD (ml/min)	78.6 (35.8)	100.5 (41.2)
eGFR–CKD EPI (ml/min)	78.4 (33.7)	76.8 (29.7)

The numbers are expressed in mean (standard deviation) format

*CKD EPI* Chronic Kidney Disease Epidemiology Collaboration, *eGFR* estimated glomerular filtration rate, *MDRD* modifications of diet in renal disease

The eGFR using MDRD formula and CKD EPI formula overestimated the GFR 9.3 and 8.1 ml/min in average in males (Additional file 1: Figure S1), while it was 23.7 and 0.04 ml/min for the females respectively. While CCr and CKD EPI have not produced any outlier data, MDRD has produced number of outliers, in both male and female subgroups, at the high GFR end.

#### Precision and bias of the estimation

**Fig. 1:**
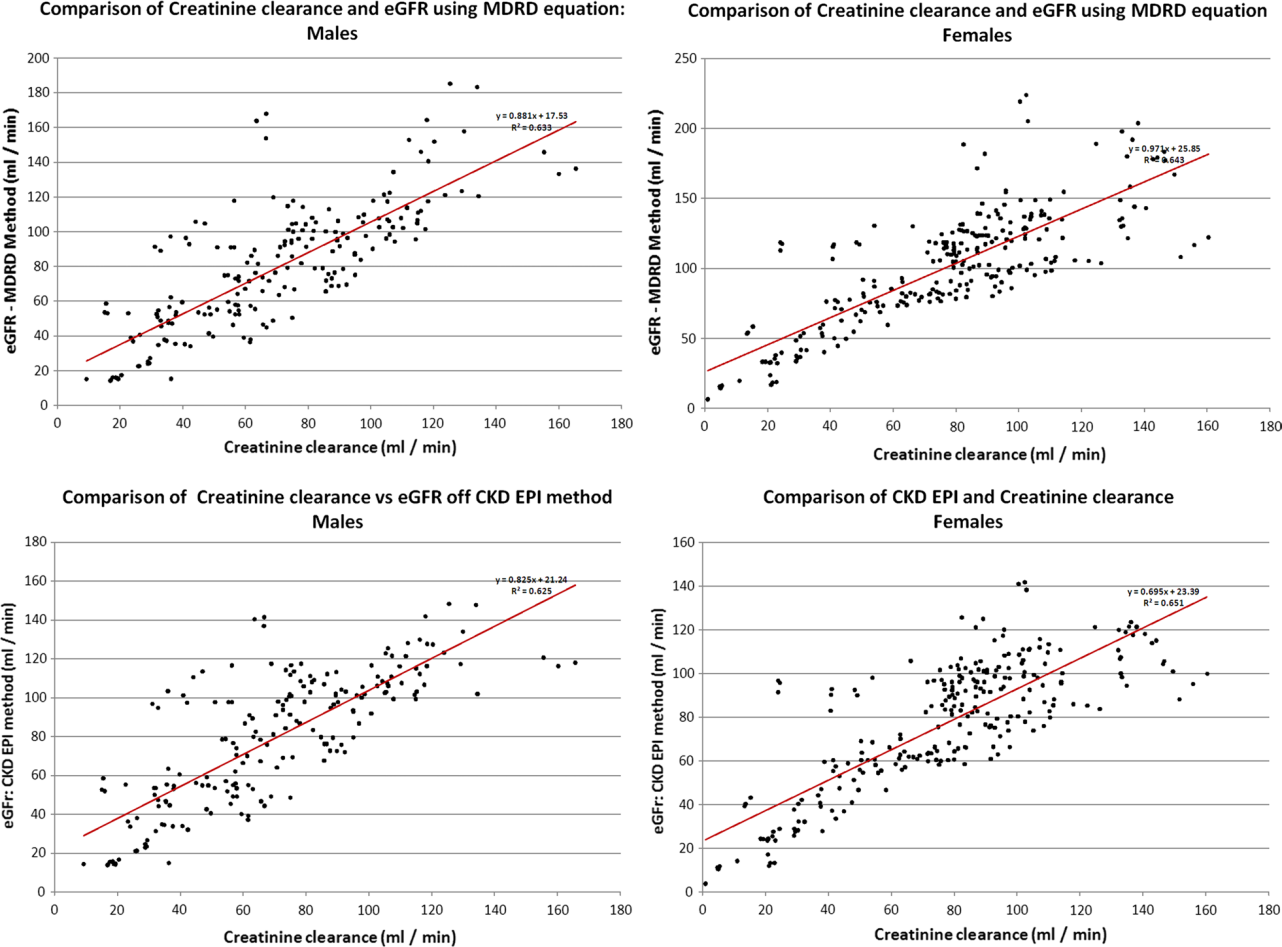
Comparison of Creatinine clearance vs renal functions estimated from (1) MDRD for males, (2) MDRD for females, (3) CKD EPI for males and (4) CKD EPI for females

We compared the two methods of estimating GFR against the CCr using linear regression. Males and females have already shown wide differences, we carried out analysis for genders separately. The results of the regression analysis are displayed in Fig. 1.

The R^2^ values for four subgroups (gender vs. MDRD/CKD EPI) was between 0.626 and 0.652, showing moderately strong relationship.

The bias and the precision of the equations, as defined in methodology section, were used to calculate CRMSE, which assesses the accuracy of the equations. Additionally data was observed for the accuracy of prediction within 15%, 30% and 50% (p15, p30 and p50) of the actual value. Table 2 summarizes the above data on accuracy, and precision of measurement.

**Table 2:** Bias and precision of each equation by gender, and accuracy of prediction at ± 15%, ± 30% and ± 50% levels (p15, p30 and p50)

Equation	Gender	N	Bias	Precision	Accuracy within subjects			CRMSE
					± 15%	± 30%	± 50%	
MDRD	Male	212	− 9.34	22.02	41.0%	68.4%	84.0%	23.92
	Female	263	− 23.65	24.97	25.5%	47.1%	71.1%	34.39
CKD-EPI	Male	212	− 9.15	21.41	40.1%	67.0%	84.4%	23.28
	Female	263	0.04	20.45	44.5%	79.5%	91.6%	20.45

*Bias* mean of the difference of estimated and measured renal functions. *Precision* standard deviation of bias, *CRMSE* combined root mean square error

In the case of males, about 40% and 65% of all cases were predicted with 15% and 30% accuracy respectively by both equations. However the difference was marked in females. CKD EPI had 44.5% and 79.5% accuracies (15% and 30% respectively) in prediction; MDRD was much poorer with it with 25.5% and 47.1%.

#### CKD staging

The study sample was heterogeneous for the KDOQI CKD stage. Out of the 263 females in the study population, 62.4% (n = 164) had their eGFR > 90 ml/min. Among males this was 44.8% (n = 95). This is statistically significant difference (Χ^2^ = 14.63, p < 0.001). Advanced CKD (Stages 4 and 5) was seen in 8.0% among males and 10.2% among females. The CKD staging performed according to MDRD show striking similarity to that done with CCr in both genders. The trend is same with CKD EPI method other than in females. CKD EPI results in a significantly lower proportion of CKD1 in females (41.1% vs. 62.2%, p < 0.0001) and significantly higher proportion of CKD 2 (14.8% vs. 33.8%, p < 0.0001). Additionally MDRD equation tend to underestimate the advanced CKD in females (10.2% vs. 5.0%, p = 0.0246) (Additional file 1: Table S2).

### Discussion

The study sample of the study has accurate representation of a cross section of patients who are presenting to an adult nephrology facility. However, our study population is different from many previous studies due to plethora of reasons. Firstly, it consists of an exclusive Asian population. Caucasian and Black patients have been evaluated in setting up [9] the equations as well as its validation [15, 16], but there have been hardly any studies [17] that involved Asian patients, and even such studies had very limited number of subjects. A recent study in Thailand [18] showed a remarkable degree of variability in CKD prevalence and risk estimates when current equations developed in Caucasians and Asians were applied to the Thai population. MDRD and CKD-EPI overestimated the prevalence of CKD two to four fold in Thai patients, and adjusted CKD EPI for Japanese, sevenfold, illustrating the fact that predictions have been quite poor. A cross-sectional study done in Pakistan [19], has demonstrated similar results to our study, with P_30_ values of 68% and 76.1% for MDRD and CKD-EPI equations respectively, and a modified CKD-EPI equation for the Pakistani subjects had better results. Similar study done with participation of Chinese subjects [20] reaffirms the findings with CKD EPI performing better than MDRD over all GFR ranges and subgroups.

Secondly, our sample had a majority of women, all of them are non pregnant, which is unique among study samples, where the highest percentage has been 53.8% [21]. Women have been traditionally under-represented despite being majority in many countries. Thirdly, the body habitus of the patients have been different to that of earlier studies. The average height (1.537 m), weight (57.7 kg) and BSAs (1.544 m^2^) all are significantly lower [9, 16, 17] than previous studies (p < 0.001).

Fourthly, our cohort is younger and has more subjects with better renal functions compared to the cohort that used to develop MDRD equation. The average CCr for males was 69.29 ml/min. and 76.84 ml/min for females. In contrast, cohort that was used to develop MDRD equation had mean GFR of 39.8 ± 21.2 ml/min, and a mean age of 50.6 ± 12.7 years. This is not surprising given the large number of potential live kidney donors that were tested in the RRL.

We used creatinine clearance as the measured GFR to compare with others. Creatinine clearance has been shown to overestimate GFR, has large inter- and intra-subject variability [9], but it was the method used as the gold standard in many of the original works. Inulin, chromium-51 EDTA or Iothalamine clearance would have been the better methods to use; sadly such resources are very costly for a nephrology unit which is specialized on transplant management. Creatinine clearance overestimates GFR about 19% [9], hence the actual GFRs should be lower than the former, and the predictive equations should return values lower than that of CCr, and we included this correction in calculating CCr for comparison with other methods that estimate GFR.

Both equations predicted an average value equal or greater than CCr, regardless of the gender. Furthermore, the CKD staging was significantly different when CKD EPI used to stage, despite it being more accurate than MDRD. The most significant difference was seen in CKD Stage 2, where the distinction between the healthy and the diseased is not very clear. The difference of the staging approaches an 20% in females in CKD Stage 2. A recent Thai study [18], shows similar results, where CKD stage changes significantly with the equation used to estimate GFR.

This is particularly important as many of the Sri Lankans are suffering from chronic interstitial nephritis of agricultural communities (CINAC) [22]. The lower protein intakes, lesser body weights, it is expected that serum creatinine to be lower than that of an age and gender matched Caucasian population. Under such situations, overestimation is inevitable. Hence the actual incidence of CKD would be much higher than eGFR based CKD staging would suggest. This theory is vindicated by change of CKD stages in statistically significant proportions when different eGFR formulae are used. Furthermore, drug dosing in renal impairment should be performed with caution, as the KDOQI CKD staging significantly changes with the method used to estimate GFR.

The R^2^ values for all the subgroups are between 0.626 and 0.652 indicating that there is a large residual factor at play. The CCr accounts for an R^2^ of 0.869 [9], when compared to Iothalamine clearance, and MDRD accounts for a R^2^ of 0.879 to 0.903 in various studies. These shows MDRD and CCr are comparable methods in estimating renal functions. We used the following equation to derive R^2^ between CCr and MDRD mathematically.

$${\text{R }} = {\text{ R}}_{ 1} {\text{R}}_{ 2} \pm \left[ {\left( { 1 { }{-}{\text{ R}}_{ 1}{^{ 2}} } \right)\left( { 1 { }{-}{\text{ R}}_{ 2}{^{ 2}} } \right)} \right]^{0. 5}$$

The term R stands for correlation between CCr and MDRD, whereas R_1_ and R_2_ stands for that of Iothalamine vs. CCr and Iothalamine vs. MDRD. This gives an estimation of 0.528 < R^2^ < 0.997, which is within the observed value of 0.626 < R^2^ < 0.656. No such information is available CKD EPI equation.

The bias and the precision and CRMSE are another ways to look at the accuracy of the equations [16]. CKDEPI had better accuracy than MDRD, especially in females. The p15, p30 and p50 values were considerably lesser than that of the comparative studies [16]. The comparative studies using mono- and multiethnic cohorts shows CKD-EPI to be a better tool for screening of CKD as well as estimating GFR [19, 23, 24].

### Conclusion

Conventional GFR estimating equations do not perform well in Sri Lankan population, and performance differs with gender. Most of them overestimate GFR. We recommend development of novel formulae to estimate GFR for the local population, using Inulin or Iothalamine clearance as the gold standard.

## Limitations

Most of the patient population is from the wet zone of the country, where the leading cause of CKD is diabetes and hypertension, and the representation of CKD of unknown origin is lesser. Secondly, we have used a method (creatinine clearance) to estimate GFR which is not regarded as the gold standard in current practice. Creatinine clearance shows intra- and inter-subject variability than inulin or iothalamine clearance.

## Supplementary information

**Additional file 1: Table S1.** Equations used to estimate GFR, BSA and CCr. **Table S2.** Summary of CKD staging, using MDRD, CKD EPI and Creatinine Clearance. **Figure S1.** Comparison of estimated glomerular filtration using Creatinine clearance, MDRD and CKD EPI equations.

**Additional file 2.** The data set of the study (SPSS Format).

## Data Availability

The datasets used and/or analysed during the current study is uploaded as a additional material (See Additional file 2).

## Abbreviations

BMI: body mass index
BSA: body surface area
CCr: creatinine clearance
CKD: chronic kidney disease
CKD EPI: chronic kidney disease epidemiology collaboration
CRMSE: combined root mean square error
eGFR: estimated glomerular filtration rate
ESRD: end stage renal disease
GFR: glomerular filtration rate
Ht: height
K/DOQI: kidney disease outcomes quality initiative
MDRD: modifications of diet into renal diseases
mGFR: measured glomerular filtration rate
NHANES: national health and nutrition examination survey
SCr: serum creatinine
UCR: urine creatinine
Wt: weight
