# Supplementary material for: Predictive performance of the estimating equations of renal function in Sri Lankan subjects
Source: BMC Res Notes. 2019 Oct 11;12:655. doi: 10.1186/s13104-019-4692-3 (PMC6788101; doi:10.1186/s13104-019-4692-3)

Additional file 1

Table S1: Equations used to estimate GFR, BSA and CCr.

| **Equation** | **Expression** | **Constants** | | |
| --- | --- | --- | --- | --- |
| DuBois | $BSA=0.01243 xw^{0.425}xh^{0.725}$ |  | | |
| MDRD | $eGFR=186 xC^{-1.154}xa^{-0.203}xK$ | Male K = 1.000  Female K = 0.742 | | |
| CKD-EPI | $eGFR=141 x\min\left( \frac{C}{K_{1}},1 \right)^{K_{2}}x\max\left( \frac{C}{K_{1}}, 1 \right)^{-1.209}x{0.993}^{a}xK_{3}$ |  | **Male** | **Female** |
|  |  | K_1_ | 0.9 | 0.7 |
|  |  | K_2_ | -0.411 | -0.329 |
|  |  | K_3_ | 1.00 | 1.159 |
| CCr | $CCr=\frac{UCrxVxBSA}{SCr x 1.73} x 0.81$ |  | | |

BSA – body surface area, eGFR – Estimated glomerular filtration rate, a – age (y), w – weight (kg), h – height (m), SCr – Serum Creatinine (mg/dl), UCr – Urine creatinine (mg/dl), K, K_1_, K_2_, K_3_– Constants, CCr – Creatinine clearance

Table S2: Summary of CKD staging, using MDRD, CKD EPI and Creatinine Clearance

| **CKD Status** | **Measured** | | **CKD EPI** | | **MDRD** | |
| --- | --- | --- | --- | --- | --- | --- |
|  | **Male %** | **Female %** | **Male %** | **Female %** | **Male %** | **Female %** |
| **1** | 44.8 | 62.4 | 45.8 | **41.1** | 42.7 | 62.6 |
| **2** | 25.9 | 14.8 | 18.9 | **33.8** | 21.8 | 20.2 |
| **3** | 21.2 | 12.5 | 26.4 | 14.4 | 27.0 | 12.2 |
| **4** | 7.5 | 7.2 | 5.7 | 6.1 | 8.1 | 3.1 |
| **5** | 0.5 | 3.0 | 3.3 | 4.6 | 0.5 | 1.9 |

Figure S1


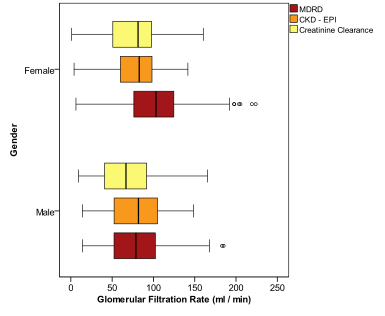

Supplement: Supplementary file 1 — Additional file 1: Table S1. Equations used to estimate GFR, BSA and CCr. Table S2. Summary of CKD staging, using MDRD, CKD EPI and Creatinine Clearance. Figure S1. Comparison of estimated glomerular filtration using Creatinine clearance, MDRD and CKD EPI equations. [file 13104_2019_4692_MOESM1_ESM.docx]
